# Quality of Sputum Specimen Samples Submitted for Culture and Drug Susceptibility Testing at the National Tuberculosis Reference Laboratory-Uganda, July-October 2013

**Published:** 2015-09

**Authors:** Lilian Bulage, Joseph Imoko, Bruce J. Kirenga, Terry Lo, Henry Byabajungu, Keneth Musisi, Moses Joloba, Emily Bloss

**Affiliations:** 1 National Tuberculosis and Leprosy Programme, Kampala, Uganda; 2 National Tuberculosis Reference Laboratory, Kampala, Uganda; 3 World Health Organization, Kampala, Uganda; 4 Division of Pulmonary Medicine, Department of Medicine, Makerere University College of Health Sciences, Kampala, Uganda; 5 Centers for Disease Control and Prevention, Atlanta, USA

**Keywords:** Tuberculosis, Sputum Specimen Quality, Uganda

## Abstract

**Setting:**

The Uganda National Tuberculosis Reference Laboratory (NTRL) in Kampala.

**Objective:**

The proportion of poor quality specimens received for drug susceptibility testing (DST) at the NTRL and factors contributing to poor specimen quality were assessed.

**Design:**

A cross-sectional study was conducted of sputum samples received at the NTRL from patients at high risk for multidrug-resistant tuberculosis (MDR TB) during July-October 2013. Demographic, clinical, and bacteriological data were abstracted from laboratory records. A poor quality sample failed to meet any one of four criteria: ≥3 milliliter (ml) volume, delivered within 72 hours, triple packaged, and non-salivary appearance.

**Results:**

Overall, 365 (64%) of 556 samples were of poor quality; 89 (16%) were not triple packaged, 44 (8%) were <3 mls, 164 (30%) were not delivered on time, and 215 (39%) were salivary in appearance. Poor quality specimens were more likely to be collected during the eighth month of TB treatment (OR = 2.5, CI = 1.2 – 5.1), from the East or Northeast zones (OR = 2.2, CI = 1.1 – 4.8), and from patients who previously defaulted from treatment (OR = 1.9, CI = 1.1 – 3.2).

**Conclusion:**

The majority of sputum samples had poor quality. Additional efforts are needed to improve quality of samples collected at the end of treatment, from East and Northeast zones, and from patients who had previously defaulted.

## 1. Introduction

Worldwide, the burden of multidrug-resistant tuberculosis (MDR TB), which is defined as TB resistant to at least isoniazid and rifampicin, is estimated to be 3.6% among newly-diagnosed TB cases and 20% among retreatment cases [1]. However, globally, and in most countries with a high burden of MDR TB, less than 25% of people estimated to have MDR TB are detected [1]. To ensure early detection of MDR TB and rapid initiation of measures to control its transmission, the World Health Organization (WHO) recommends routine testing among retreatment TB cases to rule out MDR TB [2]. However, accurate diagnosis of MDR TB requires laboratory facilities where TB culture and drug-susceptibility testing (DST) can be performed and quality control measures are in place to ensure specimens are of good quality.

In Uganda, the prevalence of MDR TB is 1.2% and 12% among new and retreatment cases respectively [3]. To improve DST services and MDR TB detection rates in Uganda, a TB Specimen Referral System (TSRS) was developed in 2009 by the National TB and Leprosy Program (NTLP) and the National TB Reference Laboratory (NTRL) for patients at high risk of MDR TB. According to national guidelines, high risk patients include all retreatment cases (*i.e*., relapse, default, failure, and chronic cases), MDR-TB contacts, and health care workers [4]. Within the TSRS, a sputum sample of at least 3 milliliters (mls) is collected by trained health care workers at local health facilities from all patients who are diagnosed with smear-positive TB and at high risk for MDR TB. The specimen is then triple packaged and transported within 72 hours of collection to the NTRL using the national postal service. Upon arriving at the NTRL, the laboratory staff evaluates each sample and documents volume, appearance, time of reception, and packaging prior to initiating culture and DST. Results are then returned to their respective health facilities through the postal service [5].

A good quality sputum sample is important in order to obtain accurate and timely DST results. However, since the establishment of the TSRS, no systematic evaluation of the quality of sputum samples arriving at the NTRL has been conducted. In response to this need and in light of a recently published systematic review highlighting the need for additional research assessing sputum quality in high TB burden settings [6], this study sought to determine the proportion of poor quality specimens received at the NTRL and assess factors associated with poor specimen quality.

## 2. Data Collection

A cross-sectional study of sputum samples received at the Uganda NTRL for routine patient care from patients at high risk for MDR TB (*i.e*., previously treated cases, including relapse, default and treatment failure, MDR-TB contacts, and health care workers) was conducted during July 24-October 24, 2013. Only samples received through the TSRS at the NTRL for programmatic purposes were included (*i.e*., samples collected specifically for other research protocols were excluded). Samples from patients with newly diagnosed TB, on MDR-TB treatment, or with extra pulmonary disease, and samples intended for quality control were excluded from the study. A pre-tested standardized data abstraction tool was used to capture demographic, clinical, and laboratory information from NTRL laboratory records, including TSRS request forms, sample reception forms, and the electronic laboratory data base.

## 3. Definitions

Based on standards set by the NTRL and WHO, information was collected on four indicators measuring sputum specimen quality: timeliness, volume, packaging, and appearance [7]–[9]. Timeliness of sample delivery was defined as the number of hours from collection of the sample from the patient at the health facility to its reception at the NTRL. Based on the laboratory standard operating procedures, transportation of the sample should not exceed 72 hours. The sample volume was deemed adequate if a sputum sample received at the NTRL for culture and DST was at least 3mls in volume. For packaging, a sputum sample was adequately packaged if it was triple packaged (*i.e*., sample packed in a falcon tube, cotton wrapped around the tube, placed in a zip lock bag, and placed in a secure shipping box). Samples that appeared muco-salivary, mucoid, muco-purulent, purulent, or blood stained were defined as sufficient, but those which appeared salivary were deemed to have a poor appearance. A composite variable was created using the above four indicators. A sputum sample was defined to be of good quality if it met each of the four quality criteria. Any sample which did not meet all of the above criteria was defined as poor quality. If the percentage of missing cases for each quality criteria was less than 5% for a given category, those cases were removed from the analysis; otherwise missing data were defined as poor quality.

## 4. Data Analysis

Data were entered into Epi-data (version 3.02) and later exported to Epi-info (version 3.5.1) for cleaning and analysis. Univariate analyses were conducted to obtain frequencies and proportions about the baseline characteristics of the samples, including the proportion of samples which met the quality requirements. Bivariate analyses were used to determine strengths of association between the independent variables and specimen quality. Crude odds ratios (OR) and 95% confidence intervals (CI) were calculated. Multivariate analysis was used to determine variables independently associated with specimen quality using adjusted odds ratios (AOR) and associated confidence intervals. Factors were entered into logistic regression models based on biological plausibility, previous literature, and statistical significance in bivariate analysis at a p < 0.05 level.

## 5. Ethics

Ethical approval was sought from the Uganda Virus Research Institute (UVRI) Science and Ethics Committee and subsequently from the Uganda National Council of Science and Technology. Additional review by the Centers of Disease Control and Prevention (CDC) institutional review board was not required because CDC investigators were determined not to be engaged in human subjects research as defined by relevant US government regulations (*i.e*., CDC investigators did not interact with study subjects or have access to identifiable data for study subjects).

## 6. Results

### 6.1. Sample Characteristics

**Figure 1.**
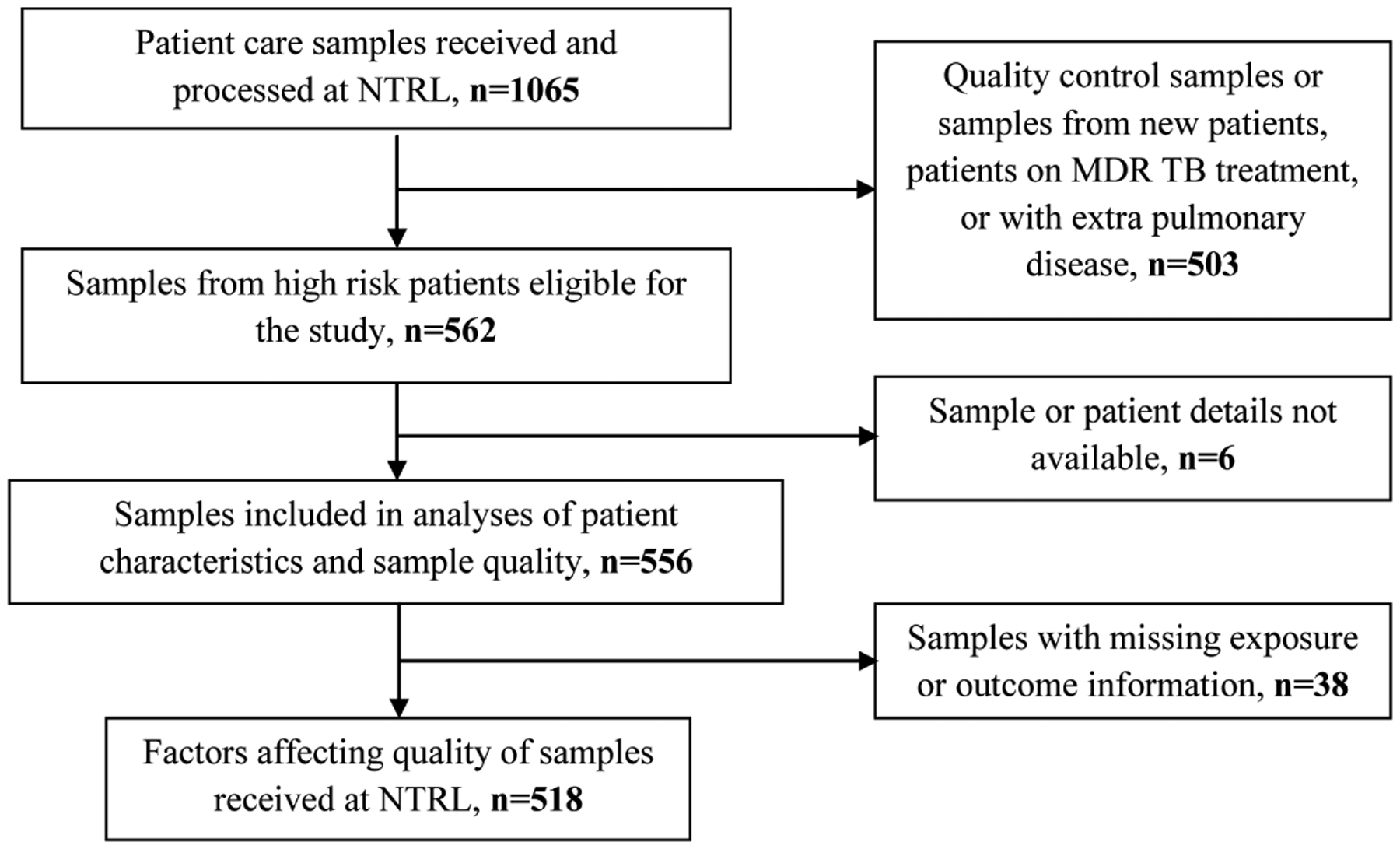
Flow chart illustrating the number of sputum samples received at NTRL during July-October 2013 and included in analysis.

Of 1199 TB diagnostic treatment units in Uganda (based on NTRL external quality assurance data, 2013), 220 (18%) facilities sent routine patient care samples to the NTRL through the TSRS during July 24-October 24, 2013 and were included in the study. Of the 1065 patient care samples received and processed during the study period, 503 were excluded because they were from patients with newly diagnosed TB or with extra pulmonary disease, on MDR-TB treatment, or they were quality control samples (Figure 1). Of the 562 samples eligible for inclusion in the study, 6 samples were excluded due to missing or incomplete samples or patient identification information.

**Figure 2.**
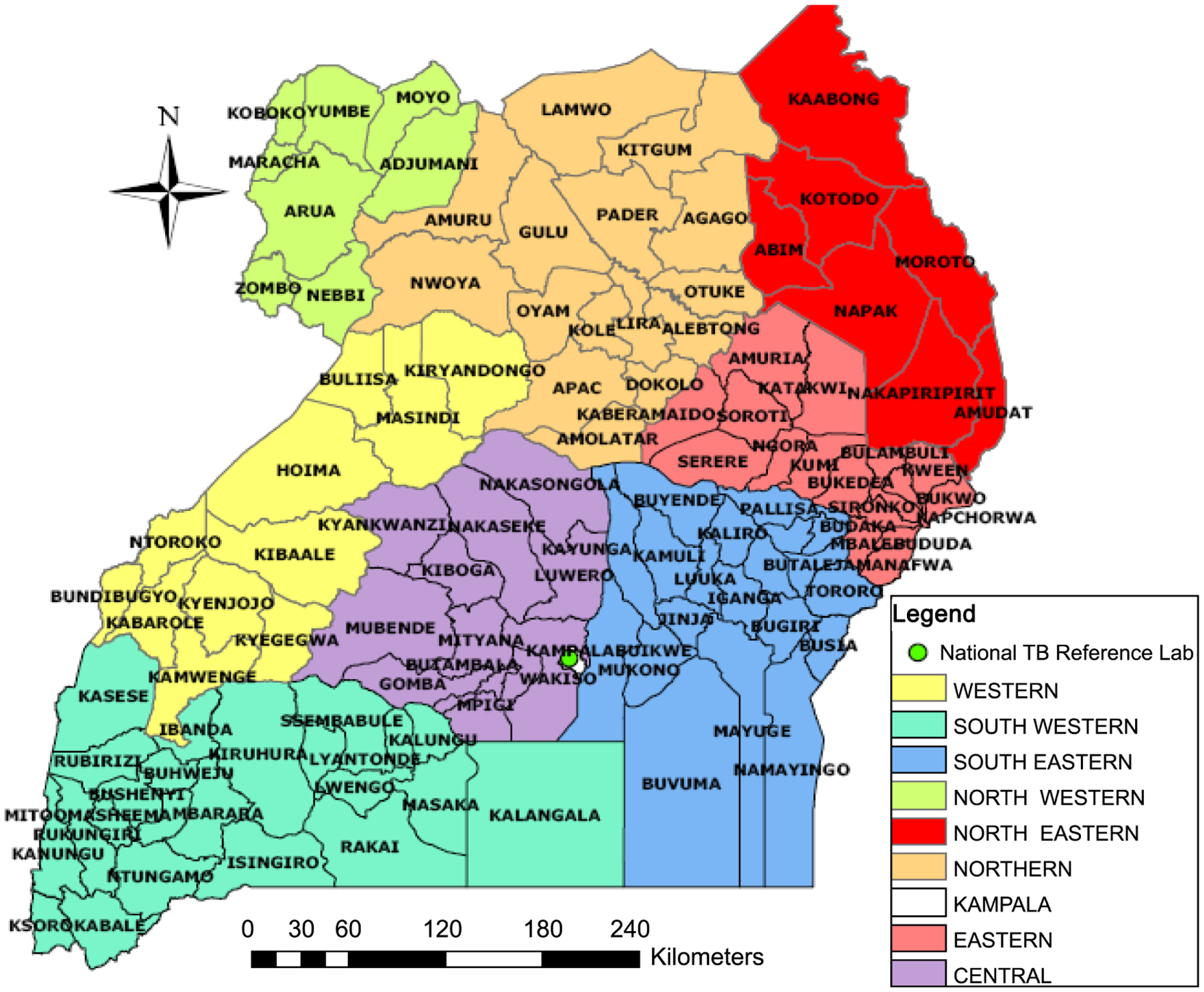
Map of national tuberculosis and leprosy program Uganda TB Zones.

Of the 556 samples included in the study, most were from patients who were male (69%), aged 31 – 40 years (30%), had failed treatment (38%) and were from regional referral hospitals (32%) in the North and Northwest zones (30%) (Table 1, Figure 2). The largest proportion of samples were collected during the eighth month of TB treatment (27%). After excluding 38 samples with missing exposure or outcome data, there were 518 samples included in the analysis of factors affecting sample quality.

### 6.2. Quality of Samples

Overall, 365 (64%) of 556 samples included in the study were of poor quality; 16% (89/556) were not triple packaged, 8% (44/556) were <3 mls, 30% (164/556) were not delivered on time, and 39% (215/556) were salivary in appearance (Table 2). In contrast, 162 (29%) of the samples were defined as good quality. Compared to samples of good quality, a greater proportion of poor quality samples were from females (32% vs 26%), patients aged 1 – 20 years (7% vs 5%), from district hospitals (20% vs 15%) and Health Center IIIs (8% and 5%), although these relationships were not statistically significant. In multivariate analysis, poor quality specimens were more likely to be collected during the eighth month of TB treatment (OR = 2.5, CI = 1.2 – 5.1), from the East or Northeast zones (OR = 2.2, CI = 1.1 – 4.8), and from patients who previously defaulted from treatment (OR = 1.9, CI = 1.1 – 3.2) (Table 3).

## 7. Discussion

In this cross-sectional study examining the frequency of and factors related to poor quality specimens in Uganda, we found that nearly two-thirds of the samples received at the national referral laboratory for culture and DST were of poor quality based on four criteria, including volume, timeliness, packaging, and appearance. This has important implications for TB control in Uganda because poor quality samples can impact the ability to detect smear- and culture-positive TB and conduct drug-susceptibility testing, which in turn delays detection and treatment of MDR TB and implementation of measures to control its transmission in the community [10]–[12].

To improve the quality of samples within the TSRS, some operational changes are needed. Namely, after all four quality criteria (volume, timeliness, packaging and appearance) are checked upon receipt of the samples at the NTRL, phone calls to health facilities referring poor quality specimens should be routinely implemented to understand challenges providers face in collecting good quality samples, to give providers immediate feedback, and to request another sputum sample from the patient, if deemed necessary. Additionally, quality criteria should be routinely documented, analyzed, and reviewed at the NTRL in order to monitor sample quality trends and geographic hot spots for poor quality specimens. Results should be disseminated at local, district, and regional laboratories as a part of routine supportive supervision and quarterly review meetings to facilitate improvement in specimen quality at the NTRL.

Sample appearance and volume are largely patient-dependent. In this study, the largest proportion (39%) of poor quality samples was due to poor appearance, while the smallest proportion was due to low volume (8%). There is a need to ensure that providers deliver sufficient, accurate health education and counseling to patients at the time of sample collection to demonstrate how to produce good quality sputum. This approach has been demonstrated to be effective in a randomized clinical trial conducted in Indonesia among presumptive TB cases [13]. Evidence suggests that, in practice, providers are often constrained by time and as a result offer limited guidance to their patients [14] [15]. Coughing should be supervised to ensure that sputum is collected correctly; unsupervised patients are less likely to provide adequate specimens [16]. Furthermore, the NTLP may adopt an efficient and standardized approach to patient health education in which health providers follow structured guidelines and provide user-friendly translated materials, including visual aids, to TB patients [17].

Timeliness and packaging are primarily related to provider practices or system deficiencies. The study revealed one-third of samples were not received within 72 hours and 16% had sub-optimal packaging. These findings highlight the importance of expanding the roll-out of more sensitive diagnostic tests for TB and drug resistance for pulmonary tuberculosis in adults to peripheral laboratories in Uganda. The Xpert MTB/RIF assay is highly sensitive and specific and can detect TB and rifampicin resistance from clinical specimens within two hours after starting the test, with minimal requirements for facilities [18]. Even though culture and DST are still needed to detect TB in acid-fast bacilli-negative samples, confirm rifampicin resistance in persons at low risk of having MDR TB and determine susceptibilities to other anti-TB drugs, this new technology can help hasten detection and treatment of persons with MDR TB in Uganda.

In this study, samples collected in the East and Northeast zones were more likely to be of poor quality. The East and Northeast zones in Uganda are characterized by hard-to-reach areas (e.g., Karamoja), where poor infrastructure, access, and utilization of basic health services challenge the effectiveness of TSRS. Additional resources need to be allocated to hard-to-reach areas to strengthen TB laboratory testing, transportation, and referral services in those regions.

In this study, samples collected from patients who had defaulted were more likely to be of poor quality. Inadequate patient knowledge about tuberculosis is a key factor affecting the quality of tuberculosis services and is associated with default [19]. User-friendly translated information, education, communication (IEC) materials may help minimize the information gap. Samples collected at 8 months were also less likely to be of good quality, which may be because of the effect of treatment on sputum.

There were several limitations to this study. First, the number of health facilities which participated in referring the samples to NTRL within TSRS was low (approximately one-fifth of the eligible facilities). While this is not a significant study limitation, it does suggest a limitation of the coverage of the TSRS. There have since been efforts by the NTRL to improve the system through partnering with non-government organizations and implementing partners, and utilizing the National HUB Specimen Transportation Network System, which was set up in 2011, among other interventions. Second, for a number of samples, the date of sample collection was not indicated, making it impossible for the timeliness indicator to be calculated. This could be attributed to the limited training and knowledge of the provider about the importance of accurately completing the laboratory request form. Despite these limitations, the study’s greatest strength was that it was a national level study which collected information on the four core quality criteria on all patient care samples received at the NTRL.

## 8. Conclusion

Since the establishment of the TSRS in 2009, no systematic documentation of its performance has been done. This study provides initial evidence that can be used to improve sputum sample quality and ultimately ensure timely and accurate diagnosis of drug-resistant TB and its control in Uganda. This evaluation found the majority of sputum samples received at the NTRL did not meet all the four quality indicators of volume, appearance, packaging, and timeliness. Samples collected at the end of treatment, from the East and Northeast zones, and from patients who had previously defaulted were more likely to be of poor quality. These findings highlight an urgent need to focus on improving specimen quality in Uganda, especially in these groups.

## Figures and Tables

**Table 1.** Characteristics of the sputum samples received at NTRL during the study period (N = 556).

Variable	Frequency (Percentage)
**Sex**	
Male	386 (69.5)
Female	167 (30.0)
Missing	3 (0.5)
**Age Category**	
1 – 20	33 (5.9)
21 – 30	138 (24.8)
31 – 40	168 (30.2)
41 – 50	126 (22.7)
51 – 89	84 (15.3)
Missing	6 (1.1)
**Type of Health Facility**	
Regional Referral Hospital	179 (32.2)
Mulago National Referral Hospital	37 (6.7)
District Hospital	141 (25.3)
Health Center IV	105 (18.9)
Health Center III	94 (16.9)
**TB Zones**	
North/Northwest	166 (29.9)
West/Southwest	108 (19.4)
East/Northeast	68 (12.2)
Southeast	70 (12.6)
Central	70 (12.6)
Kampala	74 (13.3)
**Patient Category**	
Failure	211 (37.9)
Default	130 (23.4)
Relapse	162 (29.1)
Chronic case*	40 (7.2)
MDR-TB contact	12 (2.2)
Health care worker	1 (0.2)
**Timing of Sputum Collection**	
0 months	62 (11.2)
2 months	110 (19.8)
5 months	86 (15.5)
8 months	150 (27.0)
Missing	148 (26.5)

Note: Health Center IIIs provide basic preventive, promotive and curative care. They also provide supportive supervision of the community and smaller health centers under their jurisdiction. There are provisions for laboratory services for diagnosis, maternity care, and first referral cover for the sub-county. Health Center IVs oversee all curative, preventive, promotive and rehabilitative health activities including those carried out by service providers in the health sub-district.

* Chronic cases were defined as patients who continued to be smear and/or culture-positive after the completion of a re-treatment regimen.

**Table 2.** Quality of sputum samples received at NTRL during the study period (N = 556).

Variable	Frequency (Percentage)
**Packaging**	
Triple packaged	467 (84.0)
Not triple packaged	89 (16.0)
**Volume**	
≥3 mls	505 (90.8)
<3mls	44 (7.9)
**Timeliness**	
≤3 days	313 (56.3)
>3 days	164 (29.5)
Missing	79 (14.2)
**Appearance**	
Salivary	215 (38.7)
Not salivary*	336 (61.3)
**Quality** **	
Poor quality	365 (64.0)
Good quality	162 (29.1)
Missing	38 (6.8)

Totals may not sum to N = 556 for each variable because when the percentage of missing cases was <5% for a given category, those cases were removed from the analysis.

* Mucosalivary, mucoid, mucopurulent, purulent, or blood stained.

** A good quality sample met each of four criteria: >3 ml volume, delivered to NTRL within 72 hours from time of collection, triple packaged, and not salivary in appearance. Any sample which did not meet any of the above criteria was defined as a poor quality sample.

**Table 3.** Factors associated with quality of sputum samples received at NTRL during the study period (N = 518).

Variable	Poor quality n (%)	Good quality n (%)	OR	95%CI	AOR	95%CI
**Sex**						
Male	242 (68.4)	119 (73.9)	-	-	-	-
Female	112 (31.6)	42 (26.1)	1.3	0.9 – 2.0	-	-
**Age Category**						
1 – 20	23 (6.5)	8 (5.0)	-	-	-	-
21 – 30	91 (25.9)	34 (21.3)	0.9	0.4 – 2.3	-	-
31 – 40	104 (30.0)	56 (35.0)	0.6	0.3 – 1.5	-	-
41 – 50	77 (21.9)	41 (25.6)	0.7	0.3 – 1.6	-	-
51 – 89	57 (16.2)	21 (13.1)	0.9	0.4 – 2.4	-	-
**Type of Health Facility**						
Regional Referral Hospital	111 (31.2)	51 (31.5)	-	-	-	-
Mulago National Referral Hospital	84 (23.6)	47 (29.0)	0.8	0.5 – 1.3	-	-
District Hospital	72 (20.2)	24 (14.8)	1.4	0.8 – 2.4	-	-
Health Center IV	61 (17.1)	32 (19.8)	0.9	0.5 – 1.5	-	-
Health Center III	28 (7.9)	8 (4.9)	1.6	0.7 – 3.8	-	-
**TB Zones**						
North/Northwest	106 (29.8)	47 (29.0)	-	-	-	-
West/Southwest	64 (18.0)	38 (23.5)	0.8	0.4 – 1.3	0.9	0.5 – 1.5
East/Northeast	53 (14.9)	11 (6.8)	2.1	1.1 – 1.5	**2.2**	**1.1** – **4.8**
Southeast	44 (12.4)	25 (15.4)	0.8	0.4 – 1.4	0.8	0.4 – 1.5
Central	42 (11.8)	23 (14.2)	0.8	0.4 – 1.5	0.8	0.4 – 1.5
Kampala	47 (13.2)	18 (11.1)	1.2	0.6 – 2.2	1.4	0.7 – 2.8
**Patient Category**						
Failure	125 (35.1)	68 (42.0)	-	-	-	-
Default	93 (26.1)	31 (19.1)	1.6	1.1 – 2.7	**1.9**	**1.1** – **3.2**
Relapse	101 (28.4)	48 (29.6)	1.2	0.7 – 1.8	1.3	0.8 – 2.0
Chronic case	26 (7.3)	13 (8.0)	1.1	0.5 – 1.3	0.9	0.5 – 2.0
MDR-TB contacts and health workers	11 (3.1)	2 (1.2)	3.0	0.6 – 13.9	3.8	0.8 – 18.9
**Timing of Sputum Collection**						
0 months	34 (9.6)	22 (13.6)	-	-	-	-
2 months	74 (20.8)	33 (20.4)	1.4	1.7 – 2.9	1.9	0.9 – 3.9
5 months	48 (13.5)	31 (19.1)	1.0	0.5 – 2.0	1.3	0.6 – 2.9
8 months	102 (28.7)	36 (22.2)	1.8	1.1 – 3.5	**2.5**	**1.2** – **5.1**
Missing	98 (27.5)	40 (24.7)	1.6	0.8 – 3.0	1.7	0.8 – 3.3

Numbers may not sum to N = 518 for each variable because when the percentage of missing cases was <5% for a given category, those cases were removed from the analysis. OR = unadjusted odds ratio; AOR = adjusted odds ratio.
